# Effect of root canal sealers on human periodontal ligament fibroblast viability: ex vivo study

**DOI:** 10.1007/s10266-017-0329-y

**Published:** 2017-12-14

**Authors:** Grzegorz Szczurko, Małgorzata Pawińska, Elżbieta Łuczaj-Cepowicz, Anna Kierklo, Grażyna Marczuk-Kolada, Adam Hołownia

**Affiliations:** 1 Department of Integrated Dentistry, Medical University of Białystok, M. Skłodowska-Curie Street 24A, 15-276 Białystok, Poland; 2 Department of Pediatric Dentistry, Medical University of Białystok, J. Waszyngton Street 15A, 15-274 Białystok, Poland; 3 Department of Dentistry Propaedeutics, Medical University of Białystok, J. Waszyngton Street 15A, 15-274 Białystok, Poland; 4 Department of Clinical Pharmacology, Medical University of Białystok, J. Waszyngton Street 15A, 15-274 Białystok, Poland

**Keywords:** Cytotoxicity, Flow cytometry, MTT, Periodontal ligament fibroblasts, Root canal sealer

## Abstract

The aim of the study was to compare ex vivo the toxic effects of six root canal sealers immediately after mixing or setting on human periodontal ligament fibroblasts (HPdLF). Freshly mixed (I group) or set (allowed to dry for 24 h) (II group) specimens of AH Plus Jet (AH), Apexit Plus (AP), MTA Fillapex (FL), GuttaFlow (GF), MetaSEAL Soft (META), and Tubli-Seal (TS) were prepared. HPdLF were exposed for 24 h to the specimens. 3-(4,5-dimethylthiazolo-2-yl)-2,5-diphenyltetrazolium bromide assay was used to examine the effect of the root canal sealers on mitochondrial metabolic activity. Fluorescein isothiocyanate (FITC)-annexin V (AnV) and propidium iodide staining followed by flow cytometry was used to identify the effects of the materials on cell apoptosis/necrosis. Statistical analyses were performed by one-way ANOVA followed by post hoc tests, and significance was determined at *P* < 0.05. Most materials from the two groups reduced the viability of the cultured cells compared with the control group (*P* < 0.05). Statistical analysis showed significant differences in HPdLF viability between the individual materials in each group (*P* < 0.001). AH and AP induced a significant increase in the percentage of apoptotic cells, while TS, FL, and META elevated the proportion of necrotic cells compared with other materials and the controls (*p* < 0.05). The cytotoxic effects of the tested root canal sealers (both fresh and set) on HPdLF varied. Both forms of sealers were able to cause toxic effects by inducing apoptosis and necrosis in HPdLF. The cytotoxicity of FL, META, TS was mainly associated with necrosis, while AH and AP with apoptosis.

## Introduction

Tight sealing of the root canal system requires the use of gutta-percha as the basic material and sealer. The role of root canal sealer is to bind the primary filling material with the canal wall, seal the gaps between gutta-percha and dentin, as well as to facilitate the introduction of cones into the canal space by ensuring slipperiness [1].

Several groups of sealers, which are classified based on their chemical composition, are currently available. Calcium hydroxide sealers- Apexit Plus (AP) (IvoclarVivadent, Schaan, Lichtenstein), zinc oxide eugenol sealers- Tubli-Seal (TS) (Kerr, Salerno, Italy) and epoxy resin-based sealers- AH Plus (AH) (Dentsply De Trey, Konstanz, Germany) belong to earlier generation formulations. There has been a continuous search for an ideal formulation that would meet all of Grossman’s clinical criteria [2]. New sealers, which contain methacrylic resins- MetaSEAL Soft (META) (Sun Medical, Tokyo, Japan), silicone compounds- GuttaFlow (GF) (Coltene/Whaledent, Langenau, Germany), or mineral trioxide aggregates- MTA Fillapex (FL) (Angelus, Londrina,Brasil) have been introduced.

Although obturative materials should be present only in the root canal, methods using heated, plasticized gutta-percha, which allow sealer penetration into periapical tissues [3], particularly under favorable anatomical conditions (e.g. wide apical foramen), are increasingly used. Even despite maintaining adequate caution during obturation, there still is a risk of penetration of sealer components and the products of its degradation into periapical tissues. This can elicit local inflammatory response, thus contributing to failure in treatment despite appropriate root canal debridement and disinfection [4]. Due to a long-term contact with periapical tissues, root canal filling materials should exhibit not only excellent physical and chemical properties but also biocompatibility [5, 6]. Toxic formulations can damage tissues or hinder healing of inflamed periapical structures [7].

Before introduction into clinical use, all materials must be assessed for their potential toxicity in vitro. Although this type of study does not fully reflect the behavior of these formulations in living organisms, it provides data on their potential toxic effects on cells and tissues. The advantages of such experiments include a relatively simple research technique, repeatability, the possibility of simultaneous evaluation of many materials under identical conditions, use of small amounts of tested substances, lower cost, and shorter duration of testing compared with in vivo experiments. The disadvantages include oversimplification of methodology and difficulty interpreting the results in relation to complex processes in living organisms [8].

In vitro studies in cell cultures show that some sealers can induce the expression of metalloproteinases in fibroblasts, leading to periapical tissue extracellular matrix degradation [9]; act synergistically with bacterial toxins (LPS), increasing inflammatory responses [10]; and impair macrophage phagocytosis of bacterial cells [11]. Furthermore, it was demonstrated that some sealers can inhibit cellular respiration [12] and fibroblast proliferation [13] as well as reduce the activity of alkaline phosphatase—a key enzyme involved in bone tissue formation.

In a clinical setting, the material is introduced into the root canal immediately after mixing; however, even after setting it may exert toxic effects by releasing harmful components [14, 15]. Biocompatibility should be one of the important factors influencing the choice of sealer for endodontic treatment [16].

The aim of the study was to compare ex vivo the toxic effects of selected root canal sealers immediately after mixing as well as after setting on human periodontal ligament fibroblasts (HPdLF).

## Materials and methods

### Cell culture

Human periodontal ligament fibroblasts (Cell System HPdLFClonetics™, Lonza Walkersville, Inc., Walkersville, USA) were routinely cultivated in DMEM (Dulbecco’s Modified Eagle’s Medium; Merk Life Science, Darmstadt, Germany) supplemented with 10% fetal bovine serum (FBS) (Merk Life Science, Darmstadt, Germany), 100 µg/mL penicillin, and 100 µg/mL streptomycin at 37 °C, 5% CO_2_, and 95% humidity. After reaching confluent growth, the cells were detached with 0.25% trypsin solution supplemented with 0.53 mM EDTA. Enzyme activity was stopped by adding medium with 10% FBS. The cell suspension was diluted in fresh medium, seeded onto 6- (flow cytometry) and 24-well plates (MTT assay), and incubated for 24 h.

### Materials preparation

The experiment was performed using the materials listed in Table 1. The sealers were mixed in accordance with the manufacturers’ instructions, under sterile conditions. Immediately after preparation, the materials were applied into plastic rings 5 mm (diameter) × 5 mm (height) in size to maintain equal volumes. Rings containing materials intended for setting (set samples) were stored at 37 °C, 5% CO_2_, and 95% humidity for 24 h. Fresh formulations were mixed immediately before the experiment (fresh samples). Next, both groups of materials were transferred into inserts (surface area 0.47 cm^2^; 0.4-µm pore size) (Nunc Biokom, Warsaw, Poland) separating the sealer and establishing an indirect contact with the material and the cells. Then, the inserts were placed into 24-well tissue culture plates and incubated with HPdLFs for 24 h. Four samples were prepared for each material. Inserts with a surface area of 3.14 cm^2^ (Nunc Biokom, Warsaw, Poland) placed on 6-well cell culture plates (Nunc Biokom, Warsaw, Poland) were used to assess apoptosis and necrosis based on flow cytometry. Two samples were prepared for each material. Untreated cells served as control.

**Table 1:** Compositions of materials tested for antibacterial activity

Name	Source	Active ingredients
AH Plus™ (AH)	Dentsply DeTrey GmbH, Konstanz, Germany	Bisphenol-A epoxy resin, bisphenol-F epoxy resin, calcium tungstate, zirconium oxide, silica, iron oxide pigments, dibenzyldiamine, aminoadamantane, tricyclodecane-diamine, silicone oil
Apexit® Plus (AP)	Ivoclar Vivadent AG, Schaan, Lichtenstein	Calcium salts (hydroxide, oxide, phosphate), hydrogenised colophony, disalicylate, bismuth salts (oxide, carbonate), highly dispersed silicon dioxide, alkyl ester of phosphoric acid
GuttaFlow® (GF)	Coltene/Whaledent GmbH+Co. KG, Langenau, Germany	Gutta-percha powder, polydimethylosiloxane, silicone oil, platin catalyst, zirconium dioxide, nano-silver, coloring
MetaSEAL Soft (META)	Sun Medical, Tokyo, Japan	Liquid: 4-META, HEMA, difunctional methacrylate monomers Powder: zirconium oxide, silica, hydrophilic initiator
MTA Fillapex (FL)	Angelus Ind. de Prod. Odontolόgicos S/A, Londrina–PR–Brasil	Paste A: salicylate resin, bismuth trioxide, fumed silica Paste B: fumed silica, titanium dioxide, mineral trioxide aggregate, base resin
Tubli-Seal™ (TS)	Kerr Italia S.p.A., Salerno, Italy	Zinc oxide, barium sulfate, oleo resin, oils/modifiers, thymol iodide, eugenol

### Cytotoxicity assessment

Assessment of the toxic effects of the tested materials on human periodontal ligament fibroblasts was performed using 3-(4,5-dimethylthiazol-2-yl)-2,5-diphenyltetrazolium bromide (MTT assay) and flow cytometry following cell staining with fluorescein isothiocyanate conjugated with annexin V (FITC-AnV) and propidium iodide (PI).

### MTT assay

This method enables determining cell viability and proliferation based on the mitochondrial activity of succinate dehydrogenase. In viable cells, the enzyme reduces a yellow tetrazole salt—3-(4,5-dimethylthiazol-2-yl)-2,5-diphenyltetrazolium bromide (MTT)—to a purple formazan. The dye content is determined in an absorption spectrophotometer. The amount of formazan is directly proportional to the number of viable cells in the culture. Low enzymatic activity, and thus a small amount of the purple formazan and reduced absorbance values are observed for low cell survival [15, 17–19].

Culture plates containing cells and both fresh and set materials were incubated at 37 °C, 5% CO_2_, and 95% humidity for 24 h. After this time, the inserts containing materials were removed, and 1 mL of 3-(4,5-dimethylthiazol-2-yl)-2,5-diphenyltetrazolium bromide (MTT) at a concentration of 0.5 mg/mL of medium was added, and the plates were incubated for 2 h under the above-specified conditions, in the dark. Next, the fluid was aspirated from the culture, 1 mL of isopropanol acidified with hydrochloric acid (0.04 mol L^−1^) was added, and the obtained solution was briefly stirred to dissolve formazan crystals. Absorbance was measured using Lambda EZ 201 double beam absorption spectrophotometer (Perkin Elmer, Inc. Waltham, USA) at 560 nm. Cell viability was calculated using the following formula [20]:

$$ \frac{\text{Test sample absorbance}}{\text{Control sample absorbance}} \times 100\% $$

### Flow cytometry

Flow cytometry using fluorescein isothiocyanate conjugated with annexin V and propidium iodide allows for quantitative assessment of apoptotic or necrotic cells. Annexin V bound to fluorescein isothiocyanate (FITC-AnV) is a protein, which, in the presence of calcium ions, binds specifically to cell membrane phosphatidylserine, thus allowing for the detection of early stage apoptosis. An addition of propidium iodide (PI) to the incubation mixture allows for simultaneous evaluation of cell membrane integrity. This dye does not pass through the lipid barrier, thus staining only the cells with damaged cell membranes (necrotic). PI penetrates into dead cells, where it binds to nucleic acids and when excited by blue light (*λ* = 420 nm), it emits red and orange fluorescence. Apoptotic cells emit green fluorescence by exposing phosphatidylserine and annexin on their surface. Intact (viable) cells are not stained. This test allows distinguishing 4 cell subpopulations: (1) necrotic and/or apoptotic bodies—staining only with PI; (2) late apoptotic and/or necrotic—staining with both PI and annexin V; (3) viable—no staining at all; (4) early apoptotic—staining to a varying extent with Annexin V.

HPdLFs were incubated with fresh and set materials for 24 h. After incubation, the medium was removed using a Pasteur pipette (Sarstedt, Inc., Newton, USA), and the cells were washed three times (3 × 1 mL) with buffered saline without calcium or magnesium (PBS) (Polfa Lublin, Warsaw, Poland). Periodontal ligament fibroblasts were mechanically separated from the medium.

The cells were suspended in buffer (HEPES/NaOH 10 mM, pH 7.4; 140 mMNaCl; 2.5 mM CaCl_2_) and 5 µL of Annexin V FITC and 10 µL of propidium iodide (Annexin V FITC Apoptosis Detection Kit, Merk Life Science, Darmstadt, Germany) were added. The cells were then incubated for 15 min in the dark at room temperature and analyzed using FACS Canto II flow cytometer (Becton–Dickinson, Franklin Lakes, USA) provided with filters (488 nm excitation and 633 nm emission) for the used dyes. At least 1000 counts were performed for each measurement. The experiment was repeated twice. The flow cytometer was provided by the Faculty of Pharmacy with Division of Laboratory Medicine at the Medical University of Bialystok.

### Statistical analysis

The results were expressed as mean values and standard deviation. The obtained results were analyzed statistically using Statistica 8.0 (Statsoft). One-way analysis of variance (ANOVA) supplemented with Tukey’s post hoc test (comparison between materials in different groups) and the Student’s *t* test for independent samples (comparison of materials between two groups) at a significance level of *P* < 0.05 were used.

## Results

### MTT assay

**Fig. 1:**
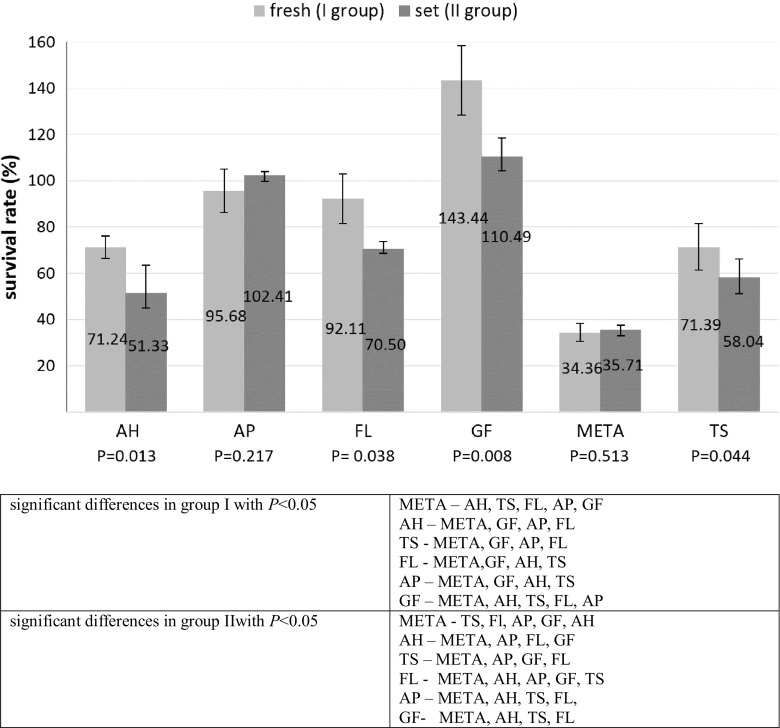
Cell viability after 24-hour exposure to fresh (I group) and set (II group) materials. Data are shown as a mean ± standard deviation. Results are expressed as a percentage of cell viability in relation to the control group. *P*-values placed under the graph indicate significant differences between fresh and set materials

The obtained results are shown in Fig. 1. Most materials from the two groups reduced the viability of the cultured cells compared with the control group. The percentage of viable cells in the group of fresh (group I) and set materials (group II) decreased in the following order: GF > AP > FL > TS > AH > META. Statistical analysis showed significant differences in periodontal fibroblast viability between the individual materials in each group (*P* < 0.001).

Both fresh and set META was significantly more toxic compared with all other sealers in both groups (*P* < 0.001). AH and TS, both immediately after mixing and after setting, were significantly more toxic than both forms of FL, AP, and GF (*P* < 0.05). Set FL was more toxic than set AP and GF (*P* < 0.001). Set AP and both forms of GF showed no cytotoxic effects on HPdLFs and even stimulated their proliferation.

Comparison of different materials in both groups showed that META exhibited significant toxicity both immediately after mixing and after setting. AH, TS, and FL were significantly less toxic in the setting form than immediately after mixing (*P* < 0.05). Detailed statistical analysis of the results is presented under Fig. 1.

### Flow cytometry

**Fig. 2:**
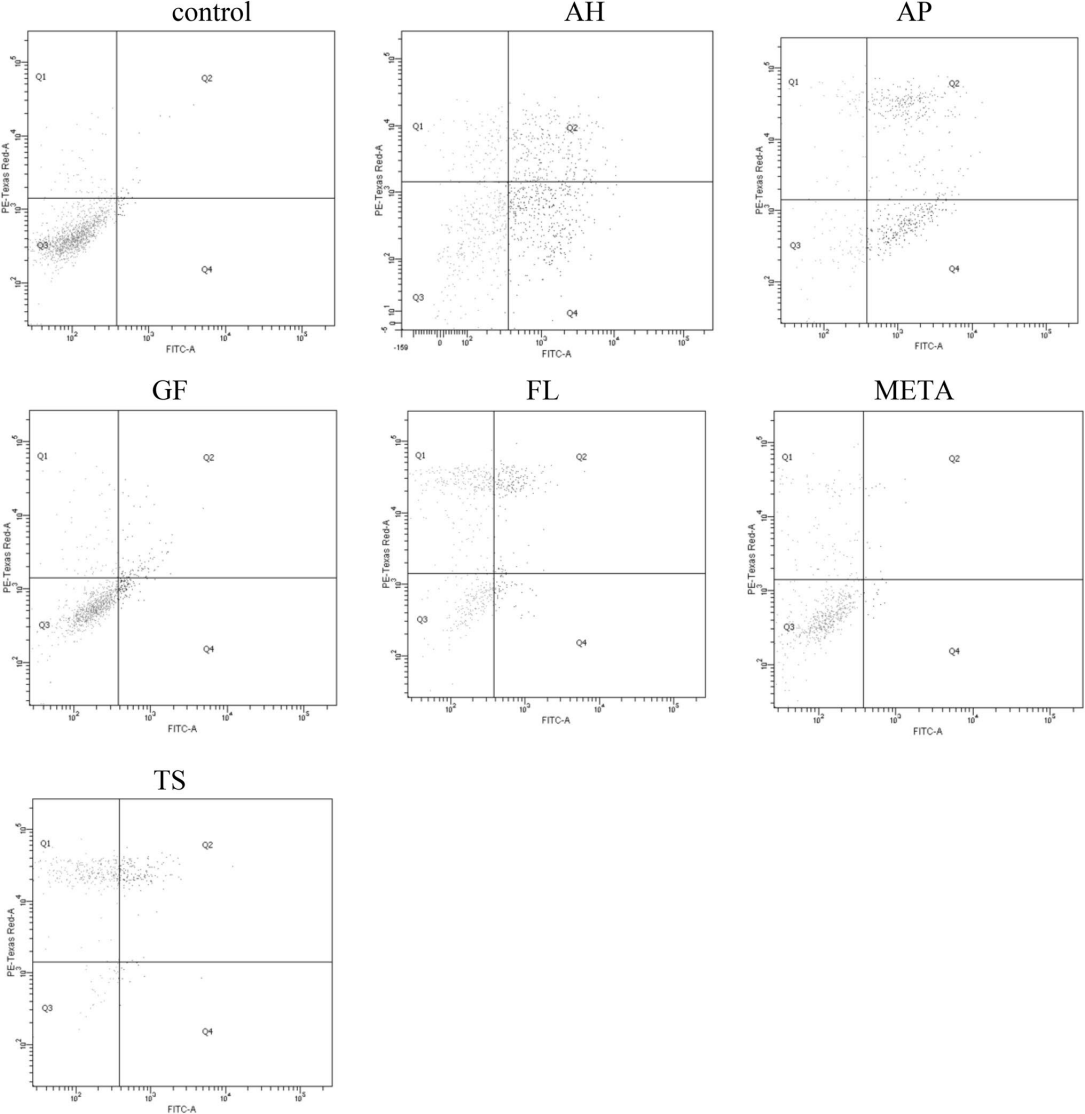
Representative two-dimensional dot plots of the flow cytometry data derived from FITC-AnV and PI-stained HPdLFs after 24-hour exposure to fresh materials. The dot plot represented the distribution of viable (lower left), early apoptotic (lower right), late apoptotic (upper right), and necrotic (upper left), respectively

**Fig. 3:**
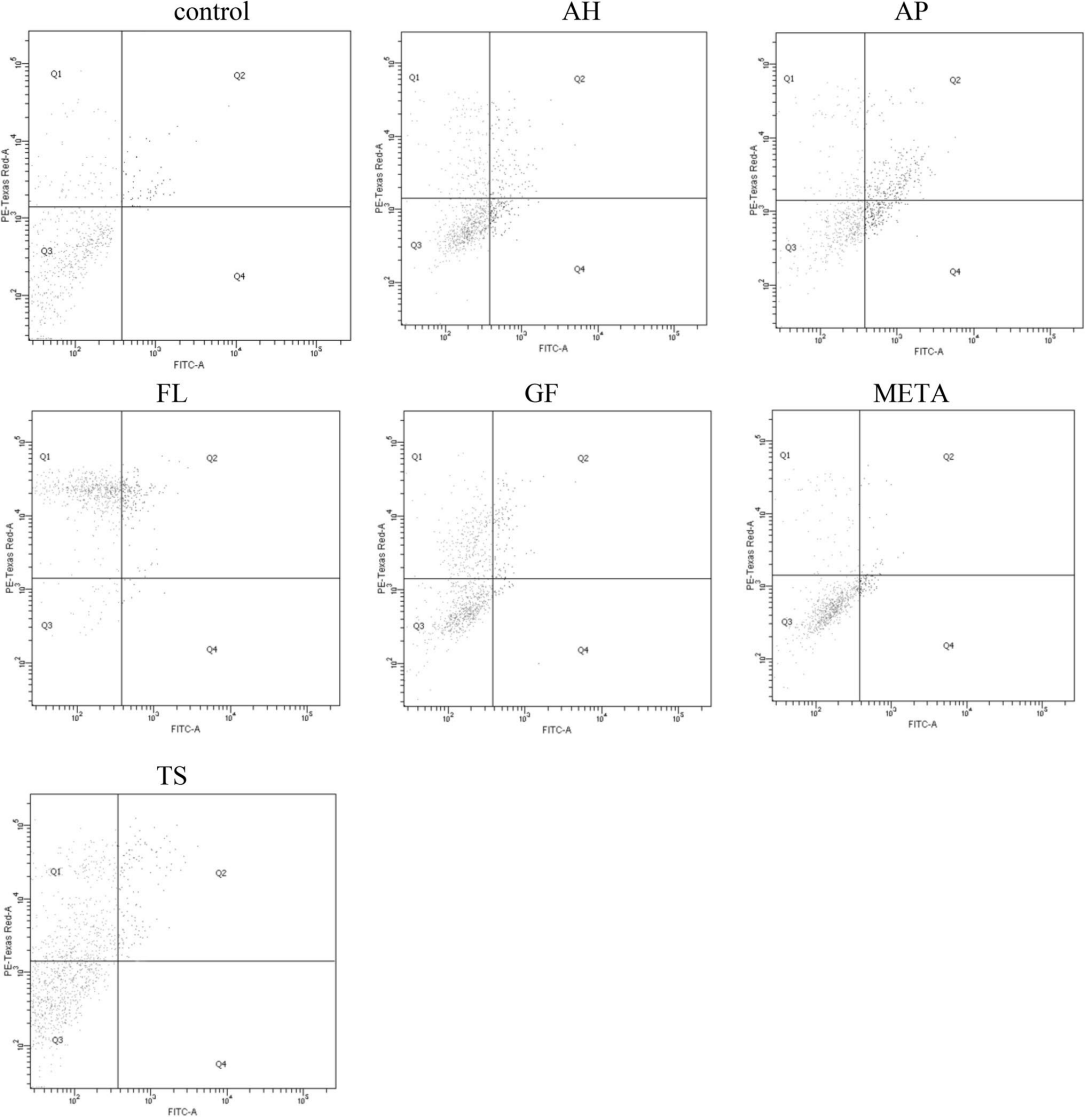
Representative two-dimensional dot plots of the flow cytometry data derived from FITC-AnV and PI-stained HPdLFs after 24-hour exposure to set materials. The dot plot represented the distribution of viable (lower left), early apoptotic (lower right), late apoptotic (upper right), and necrotic (upper left), respectively

**Fig. 4:**
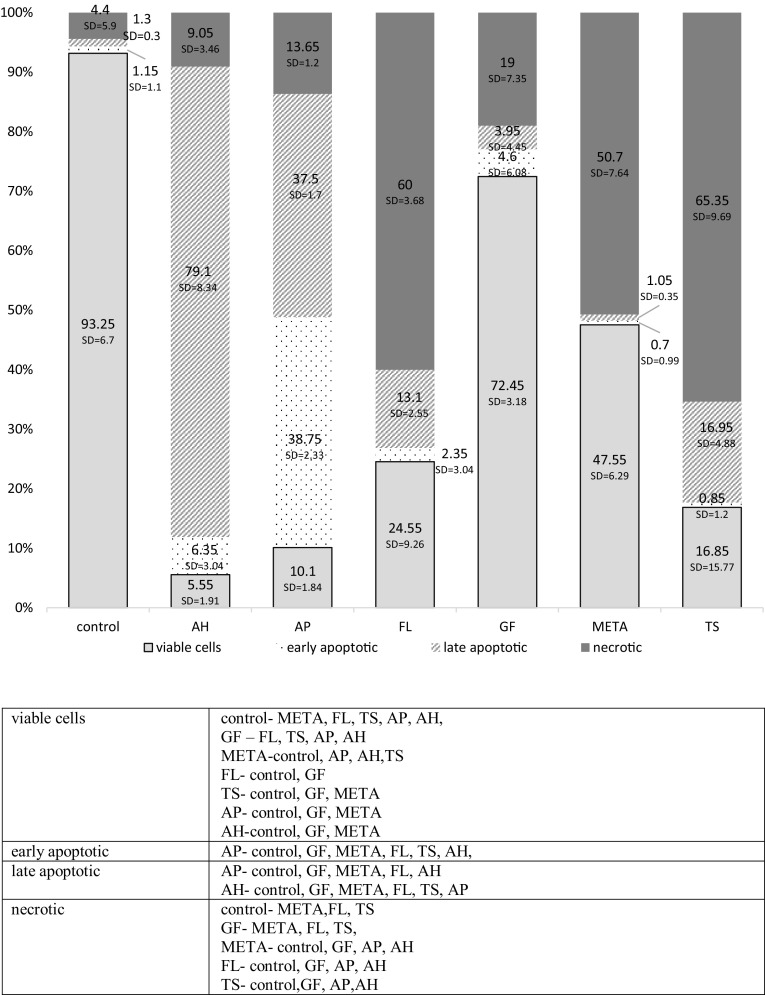
Effects of fresh materials on the viability of HPdLFs assessed using flow cytometry. The cytotoxicity was determined based on a comparison between the proportions of apoptotic and necrotic cell fractions, following the exposure of HPdLFs to the tested materials. The cumulative diagram shows the percentage of necrotic, early and late apoptotic, and viable cells (with standard deviation; SD). Significant differences with *P* < 0.05 after exposure of HPdLFs to fresh materials within the following cell population

**Fig. 5:**
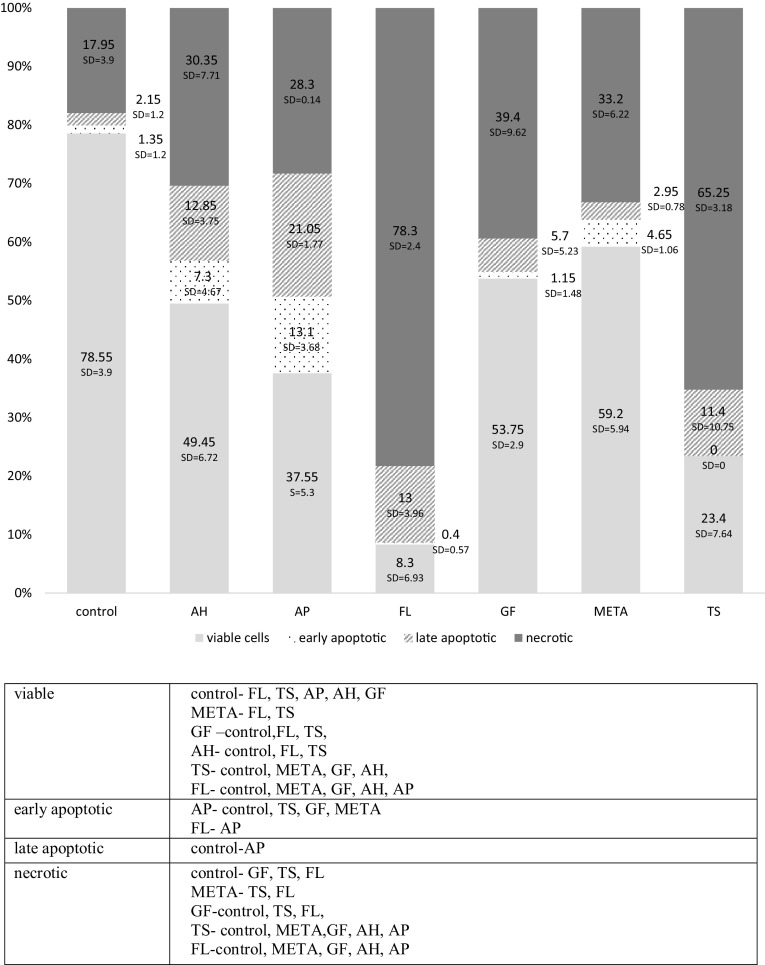
Effects of set materials on the viability of HPdLFs assessed using flow cytometry. The cytotoxicity was determined based on a comparison between the proportions of apoptotic and necrotic cell fractions, following the exposure of HPdLFs to the tested materials. The cumulative diagram shows the percentage of necrotic, early and late apoptotic, and viable cells (with standard deviation; SD). Significant differences with *P* < 0.05 after exposure of HPdLFs to set materials within the following cell population

The results are shown in the form of exemplary dot plots (cytograms)—Figs. 2 and 3, and diagrams—Figs. 4 and 5.

### Group I: fresh materials

The 24-hour exposure of HPdLFsto fresh materials resulted in a significant reduction in the percentage of viable cells in nearly all groups of cells exposed to sealers (except for GF) compared with the control group (*P* < 0.05). The lowest percentage of living cells was observed for AH, AP, and TS, with no significant differences between them (*P* > 0.05). AH and AP induced a significant growth in the percentage of apoptotic cells, while TS, FL, and META increased the proportion of necrotic cells compared with other materials and the controls (*P* < 0.05). The lowest cytotoxicity, comparable with the controls (*P* > 0.05), was shown by GF, for which cell survival was the highest. Detailed statistical analysis of the results is presented under Fig. 4.

### Group II: set materials

The 24-hour incubation of HPdLFs with set materials resulted in a significant reduction in the proportion of viable cells compared with the control group, following the use of nearly all formulations (except for META) (*P* < 0.05). The lowest cell survival was reported for FL, and was significantly compared with other sealants (*P* < 0.05), except for TS, for which comparable values were observed (*P* > 0.05). The highest percentage of viable cells, similar to that in the control group (*P* > 0.05), was observed for META.

AP induced a significant growth in the percentage of apoptotic cells, while TS and FL increased the proportion of necrotic cells in the culture compared with the other materials and the controls (*P* < 0.05). Detailed statistical analysis of the results is presented under Fig. 5.

### Comparison between fresh and set materials

Comparison between fresh and set materials showed no significant differences in cell survival following exposure to GF, META, FL, or TS sealers (*P* > 0.05). Significant differences (*P* < 0.05) were observed for AH and AP, with higher cellular mortality in the set group. Furthermore, AH and AP generated a significantly higher percentage of apoptotic cells immediately after mixing rather than setting (*P* < 0.05).

No significant differences were observed in the population of necrotic cells between fresh and set materials (*P* > 0.05).

## Discussion

The present study used reference cultures of human periodontal ligament fibroblasts to mimic clinical conditions. To exclude the risk of mechanical injury to the cells caused by the material, the preparations were placed on semi-permeable membrane inserts, imitating proper root canal filling up to the level of the apical foramen. The toxic potential of the materials was evaluated both, during their hardening as well as after setting, since it has been demonstrated based on a review of the literature that both these forms of sealers may have harmful effects on cells [21–23].

According to PN-EN ISO 10993-5/2009 for the assessment of medical products in terms of quantitative evaluation of cell viability, it is recommended to use the neutral red assay (NR); MTT assay, or its variation, the XTT assay; as well as the colony-forming assay [20]. In the experiment, MTT assay was used, which involves the measurement of mitochondrial activity of succinate dehydrogenase, thus informing about the proper functioning of the cultured cells [6, 15, 17, 19, 24]. Although the technique is simple, it is sufficiently precise and allows for obtaining quick results. The XTT assay is a modified MTT assay that eliminates the need to dissolve formazan crystals in organic solvents. The XTT assay uses a 2,3-bis(2-methoxy-4-nitro-5-sulfophenyl)-2H-tetrazolium-5-carboxanilide sodium salt, which is transformed into a product soluble in aqueous medium [25]. The Colony-Forming Ability Assay determines the proliferative capacity of cells.

### MTT assay

META showed the highest cytotoxicity. The percentage of viable cells in fresh and set groups was 34.36 ± 3.26% and 35.71 ± 1.98%, respectively. Significant toxicity of materials containing methacrylate resins was also reported by other authors [26, 27]. Garza et al. [26] assessed the effects of material eluates on L929 murine fibroblasts using the MTS assay, which is a different version of the MTT assay, where the product of dehydrogenase-mediated conversion of tetrazolium salt occurs in the presence of PMS (phenazine methosulfate) and is fully soluble in water. After the use of fresh and set META, the authors observed 10.6 ± 0.73% and 24.9 ± 7.9% of viable cells, respectively. Morrison et al. [28] investigated the survival of human periodontal ligament fibroblasts following the use of different concentrations of material eluates, based on crystal violet cell staining and the CyQUANT Cell Proliferation Assay. The authors found that the toxic effects of META can persist for up to 21 days. An experiment conducted by Yamanaka et al. [29] supported the toxic effects of META also under in vivo conditions. The material induced subcutaneous inflammatory reactions in Wistar rats, which decreased with time, but were observed up to day 28 of the experiment. Macrophages with a relatively small number of lymphocytes and neutrophils were predominant in the subcutaneous tissue contacting the sealer.

In the present experiment, lower cytotoxicity, both in fresh and set form, was showed for AH epoxy material (71.24 ± 7.45% and 51.33 ± 8.54% of viable cells in the culture, respectively). AH toxicity is attributed to the transient release of formaldehyde, which is a side product of the reaction initiating the bonding process of the material and, to a lesser extent, to amines added to the preparation to accelerate polymerization [30]. Similar findings (about 75% of living cells) were obtained by Al-Hiyasat et al. [27], who investigated the effects of AH eluates on Balb C 3T3 murine fibroblasts after 48 h of incubation using the MTT assay. In the current study, stronger toxicity of the set sealer was noticeable. Konjhodzic-Prcic et al. [31], who assessed the survival of L929 murine fibroblasts, observed a similar tendency in the behavior of cells incubated with the material. The authors found no damaging effects of AH in the first day, but they observed a decrease in the percentage of living fibroblasts to a level of 73.4% after 48 h. Adverse effects on human gingival fibroblasts persisting for 7 days were also observed by Candeiro et al. [18]. Different results were obtained by other authors. Scelza et al. [32] assessed the long-term effects of endodontic materials on human gingival fibroblasts using the MTT assay. AH induced high toxicity in the first day post incubation; the toxic effects significantly decreased after 7 and 14 days, while no significant differences in cell survival between the evaluated materials (GuttaFlow, Real Seal, AH Plus, ThermaSeal Plus, Sealapex, Roth Root 801) were observed on days 21 and 28. These divergent results may be due to the variations in experimental conditions, such as the various manners of sample preparation, the cell type, the cell material contact method, and exposure time. These factors strongly affect in vitro findings [33–35]. Silva et al. [34] also found lower cytotoxicity of AH Plus after setting. It might be caused by usage of a 3D cell culture (Balb/c 3T3 fibroblasts) and an in vitro root model. Moreover, the authors claimed that endodontic sealers have higher cytotoxic effects in the 2D cell culture model than the 3D cell culture model because of the extensive cell–cell and cell-to-matrix interactions occurring in the 3D cell aggregates and the decreased capability of sealer extracts to penetrate within the 3D cell aggregates.

Fibroblast survival similar to that for AH was observed for TS (fresh 71.39 ± 7.07%, set 58.04 ± 7.77%). The toxicity of zinc oxide eugenol sealers is mainly associated with the content of eugenol [36]. Chang et al. [14] reported that despite the fact that TS showed some cytotoxicity towards periodontal fibroblasts, it caused a transient increase in the activity of succinate dehydrogenase in the cells. The authors believe that this indicates the possible existence of adaptation mechanisms to certain irritants in fibroblasts. Huang et al. [20] noticed that cytotoxicity of materials in the same chemical group may vary considerably depending on the type of formulation used. The authors determined the cytotoxicity of three zinc oxide eugenol-based sealers (Canals, Endodmethasone and N2) on human periodontal ligament cells (PDL) and V79 cells derived from a Chinese hamster by means of MTT assay. They showed that N2 was significantly more toxic than the other sealers in both culture. Moreover, PDL cells were more sensitive to Canals than V79 cells. In contrast, endomethasone significantly inhibited V79 cell viability compared to PDL cells. Additionally, Chang et al. [14] evaluated the cytotoxicity Canals and Tubli-Seal to periodontal ligament fibroblasts (PDL) by means of MTT assay. Canals showed severe but Tubli-Seal showed moderate cytotoxicity. The authors suggest that some ingredients of Tubli-Seal (e.g. oleoresin) may modify its toxicity but more studies are necessary to elucidate this issue.

MTA Fillapex is a relatively new sealer containing mineral trioxide aggregate. Although MTA is one of the most biocompatible components [33, 37], the formulation showed some toxicity, particularly after setting (70.50 ± 2.45% of viable cells). A high solubility of MTA Fillapex after setting and leaching of the toxic substances as a result of material degradation could be contributed to the higher cytotoxicity of MTA Fillapex in the set state, than in the fresh one in our experiment [38] In the study by Mestieri et al. [6], who applied similar experimental conditions to the present experiment (the samples were kept during 24 h after mixing, MTT assay) the cell viability of MTA Fillapex ranged from 50 to 80% depending on the concentration of extracts, and this is consistent with our results. In an experiment conducted by da Silva et al. [34], the authors mimicked clinical conditions by preparing and filling dental root canals using a single gutta-percha cone technique with the evaluated sealers. Next, the filled roots were immersed in tubes containing a three-dimensional culture of Balb/c 3T3 murine fibroblasts for 24 h. Cell viability was determined using the MTT assay, showing significantly higher toxicity of MTA Fillapex (65% of living cells in the culture) compared with the control group. Other authors using the same assay showed that the toxic effects of formulation eluates on human periodontal ligament stem cells (hPDLSCs) can persist for 72 h [15]. On the other hand, in the study by Scelza et al. [39], MTA Fillapex and other materials tested (Sealapex, Pulp Canal Sealer EWT, and Real Seal) had high cytotoxic levels for human primary cells, mostly on a time-dependent basis, as shown by three different cell viability tests (mitochondrial activity -XTT, membrane integrity -neutral red test and total cell density -crystal violet dye exclusion test). However, the authors emphasized that the choice of osteoblasts could contribute to obtain such results. This kind of cells might be more sensitive than others to the cytotoxic substances derived from the sealers. Gomes-Filho et al. [40] performed an in vivo assessment of subcutaneous responses in rats implanted with polyethylene drains filled with MTA-based formulations (Endo-CPM-Sealer and MTA Fillapex). The authors observed moderately increased inflammation 7 days after the experiment, but found no inflammatory cells in the region of the implanted sealers after 60 and 90 days. Furthermore, histochemical analysis revealed the presence of granules containing calcium carbonate crystals in the region of the implanted materials. The authors believe that this indicates the biocompatibility of these sealers as well as their ability to stimulate the mineralization processes.

In this study, AP showed no toxicity; the number of living cells was 95.68% ± 8.62 (fresh) and 102.41% ± 2.01 (set). Very similar results were obtained by Konjhodzic-Prcic et al. [31], who assessed the effects of AP on L929 murine fibroblasts. The mean percentage of viable cells after the use of fresh sealer was 94.57% ± 23.83 after 24, 48 h, and 7 days.

The lowest cytotoxic effects were shown by polysiloxane-based material known as GF. The percentage of viable periodontal ligament fibroblasts was higher compared to the control group (143.44% ± 12.84 for the fresh form, 110.49 ± 6.02 after setting), which may indicate the ability of a formulation to stimulate cellular proliferation [41]. Different results were obtained by Konjhodzic-Prcic et al. [42], who observed only minor cytotoxicity (84.4% of viable cells) on day 7 of follow-up. The authors suggest that this may be associated with silver particles, which were added as a preservative, or with an incomplete chemical reaction between GF components [43]. Nevertheless, the material was classified, along with AP and AH, as low toxicity, as opposed to methacrylate-based EndoREZ (50.1% of viable cells after 7 days) [42]. Most publications support the present findings; and silicone-based materials show in vitro cell survival similar to that in controls [31, 44, 45].

### Flow cytometry

In this study, fibroblast necrosis was mainly induced by FL, TS (both forms), and fresh META. The cytotoxic effects of FL were also documented by other researchers [7, 15, 46]. Zhou et al. [46] assessed, using flow cytometry, the effects of different concentrations (1:2, 1:8, 1:32, 1:128) of fresh and set sealer extracts on human gingival fibroblasts. The highest cell death was observed at high concentrations (1:2 and 1:8) of set FL throughout the 4-week experiment. The authors also found that extracts from freshly mixed AH Plus were severely toxic, and extracts from set AH Plus of two weeks and older were no longer toxic. These results correlate with our study in relation to the fresh samples (5.5% of viable cells) but are in disagreement with respect to the set samples (almost 50% of viable cells). These discrepancies could be caused by the longer time of material setting (four weeks) in the study by Zhou et al. [46]. Rodriguez et al. [15] used Hoechst 33342 fluorescent dye to stain the DNA in human periodontal ligament stem cells (hPDLSCs) incubated with eluates of the tested materials. The process of apoptosis was identified based on the density or fragmentation of the stained nuclei using fluorescence microscopy. A significantly higher proportion of apoptotic cells vs. the control group was observed after 24, 48 and 72 h of incubation with FL eluates at 1:1 and 1:2 concentrations. Adverse effects of FL sealer can be due to silica and salicylate resins, which improve the material’s consistency, ensuring fluidity or reducing setting time [15].

META monomer contains 4-META (4-methacryloyloxyethyl trimellitate anhydride) and HEMA (2-hydroxyethyl methacrylate) resins, which have toxic effects [47]. It has been suggested that methacrylate monomers can interfere with the stable oxidation–reduction balance. By oxidation, they cause cellular oxidative stress, and thus damage cells [47]. This hypothesis indicates that methacrylate monomers induce mutations as a result of generating higher ROS levels [47]. Furthermore, HEMA induces chromosomal aberrations, thus contributing to DNA strand breakage [48].

A high percentage of necrotic fibroblasts was also found in the TS group, whose toxicity was previously confirmed using the MTT assay [20]. It should be noted that despite the adverse effects of eugenol [35, 49], zinc oxide eugenol sealers have anti-inflammatory activity. Molecular biology-based studies (PCR-polymerase chain reaction) on human dental pulp stem cells showed that eugenol down-regulated the expression of the mRNA genes responsible for the synthesis of proinflammatory cytokines (IL-1, IL-6, IL-8) [49].

Significantly, higher apoptosis rates were induced only by two of the tested materials: AH and AP, usually in the fresh form. The total percentage of early and late-stage apoptotic cells was 85.45% (AH) and 76.25% (AP). Similar AH results were obtained by Bojar et al. [50]. AP showed no cytotoxicity in the MTT assay, while the assessment of apoptosis/necrosis revealed a very low percentage of viable HPdLFs with a significant increase in the number of apoptotic cells. The induction of apoptosis may be associated with calcium hydroxide contained in the formulation and the pro-apoptotic activity of calcium ions, as confirmed by Onishi et al. [51].

The necrotic process leads to cellular membrane disruption and a release of cell contents into tissues, which stimulates granulocyte migration. Accumulation of neutrophils and the release of enzymes and reactive oxygen species increase the inflammatory response [51]. As a consequence of the increased number of apoptotic cells, modification of the inflammatory response occurs leading to changes in periapical tissues and affecting the healing process.

The current experiment indicated that the cytotoxic effects of the tested root canal sealers (fresh and set) on HPdLFs varied. Both forms of sealers were able to cause toxic effects by inducing apoptosis and necrosis in periodontal ligament fibroblasts. The cytotoxicity of FL, META, and TS was mainly associated with necrosis, while in the case of AH and AP with apoptosis. Due to the risk of persistent root canal sealer cytotoxicity, endodontic treatment should be performed in accordance with the principles that enable avoiding contact between the material and periapical tissues.
